# Cariprazine in Bipolar Disorder and Substance Use: A Dual Approach to Treatment?

**DOI:** 10.3390/ph17111464

**Published:** 2024-10-31

**Authors:** Simone Pardossi, Alessandro Cuomo, Despoina Koukouna, Mario Pinzi, Andrea Fagiolini

**Affiliations:** Department of Molecular Medicine, School of Medicine, University of Siena, 53100 Siena, Italy; alessandro.cuomo@unisi.it (A.C.); koukounadespoina@gmail.com (D.K.); mario.pinzi@student.unisi.it (M.P.); andrea.fagiolini@unisi.it (A.F.)

**Keywords:** bipolar disorder, cariprazine, substance use disorder

## Abstract

Bipolar disorder (BD) is characterized by recurrent episodes of mania, hypomania, and depression and is often complicated by comorbid substance use disorders (SUDs). Up to 60% of individuals with BD experience SUDs, which exacerbate mood instability and increase the risk of rapid cycling, suicide, and poor clinical outcomes. Current treatment strategies, including lithium and valproate, show limited efficacy in treating both BD and SUD. Psychotherapeutic approaches such as cognitive behavioral therapy (CBT) offer benefits but lack a specific focus on substances such as cannabis and cocaine. Since there is still debate on how to treat this comorbidity, there is a need to find new therapeutic options; this mini-review examines the pharmacological properties of cariprazine and its emerging role in the treatment of comorbid BD and SUD. Cariprazine, an atypical antipsychotic with partial agonism at dopamine D2 and D3 receptors, has shown promise in treating both mood symptoms and cognitive dysfunction in BD. Its unique affinity for D3 receptors, which are involved in motivation and reward processing, may offer advantages in reducing drug craving. Clinical trials indicate that cariprazine effectively treats manic, depressive, and mixed episodes in BD with a favorable side effect profile, particularly at lower doses. Preliminary results suggest its potential to reduce craving and substance use in individuals with co-occurring BD and SUD. Therefore, cariprazine, with its unique pharmacodynamic mechanism, could be further studied for the treatment of BD in comorbidity with SUD. However, evidence on the role of cariprazine in the treatment of SUDs remains limited, based primarily on case reports and animal studies. Further research, including large-scale clinical trials, is needed to determine its full efficacy in this dual diagnosis.

## 1. Introduction

Bipolar disorder (BD) is characterized by recurrent episodes of mania, hypomania, and depression, with mood instability as a defining feature. This cyclical pattern of mood disturbance contributes to significant psychosocial impairment and reduced quality of life. One of the most critical aspects of managing BD is addressing comorbidities, particularly substance use disorder (SUD), which is prevalent in this population [1]. Research suggests that between 40% and 70% of individuals with BD have a history of SUD, with comorbidity being particularly prevalent among men [2,3]. Studies suggest that up to 60% of people with BD will develop a SUD at some point in their lives, with alcohol, cannabis, and stimulants being the most commonly abused substances [4].

The predisposition to SUDs in individuals with BD can be attributed to several psychopathological features. Elevated impulsivity, sensation-seeking behaviors, and a diminished sense of risk during manic or hypomanic episodes often drive individuals towards substance use [5,6]. Conversely, depressive episodes may prompt substance use as a form of self-medication, aimed at alleviating dysphoric mood and anxiety [5]. Another key factor is stress sensitization, wherein repeated mood episodes increase sensitivity to stressors, thereby heightening the risk of relapse into substance use [6]. Additionally, impaired executive function and decision-making, often associated with prefrontal cortical dysfunction, diminishes the ability of individuals with BD to control impulsive behaviors, further complicating efforts to abstain from substances [6]. Moreover, alterations in the reward system, particularly within the dopaminergic pathways, play a critical role. These changes may enhance the drive for rewarding stimuli, such as drugs and alcohol, increasing the vulnerability to addiction [5]. Genetic factors also contribute, with evidence pointing to overlapping genetic susceptibilities for BD and SUDs, which could increase the likelihood of developing both conditions concurrently [7].

The impact of SUDs on the clinical course of BD is profound, leading to earlier onset of mood episodes, increased frequency of mood cycling, and significantly higher rates of suicide attempts [8,9]. In addition, individuals with BD who have a co-occurring SUD are more likely to experience rapid cycling and mixed episodes, which complicates the clinical management of the disorder [10]. This dual diagnosis also correlates with more severe manic and depressive episodes and greater overall illness burden [11]. The presence of a SUD is associated with increased impulsivity, which not only exacerbates mood instability but also increases the risk for further substance abuse, creating a vicious cycle that is difficult to break [12]. Gender plays a significant role in the risk of developing a SUD in individuals with BD: men are particularly vulnerable, with a meta-analysis showing that male gender is a significant predictor of SUDs in this population [3]. In addition, the number of manic episodes experienced by an individual is directly related to the likelihood of developing a SUD, with each manic episode increasing the risk [3]. Another critical factor in the relationship between BD and SUDs is the age of onset of BD: individuals with an early onset of BD are at increased risk of developing SUDs, likely due to the longer duration of illness and the increased likelihood of experiencing multiple mood episodes over time [13]. Childhood adversity, including trauma and family instability, exacerbates this risk, leading to a more severe clinical course and poorer long-term outcomes [14]. Further complicating the treatment landscape, SUDs in individuals with BD are associated with a range of adverse outcomes, including poorer treatment adherence, increased hospitalization rates, and a higher likelihood of legal and social problems [15].

Pharmacological strategies for the treatment of BD and SUDs may typically involve several classes of medications; lithium, for example, has been observed to reduce the frequency of manic and depressive episodes, which may in turn reduce the propensity to use substances as a form of self-medication [16]. Valproate has been suggested to be effective in patients with BD who also abuse substances such as cannabis or cocaine, although the evidence is inconclusive [17]. However, research supporting these pharmacological interventions is often limited by small sample sizes and methodological challenges, such as lack of randomization and blinding, which undermine the generalizability of the findings [8]. In addition to mood stabilizers, other pharmacological agents have been explored: citicoline, a neuroprotective agent, has shown promise in reducing cocaine use in BD patients in preliminary studies [18]. Similarly, the use of antipsychotics such as quetiapine and risperidone has been investigated for their potential dual benefit in stabilizing mood and reducing craving, but results have been mixed, with some studies showing significant reductions in substance use and others reporting no substantial effects [19].

Psychotherapeutic interventions may be a critical component of treatment: integrated treatment models that combine elements of cognitive behavioral therapy (CBT), motivational interviewing, and contingency management have been shown to be more effective than treatments that focus on BD or SUD alone [20]. However, while these integrated therapies have demonstrated efficacy, there remains a notable gap in the literature regarding psychotherapeutic interventions specifically targeting illicit drug use in BD. Most research has focused on alcohol use disorder, leaving a critical need for studies focusing on other substances such as cannabis, cocaine, and opioids [21].

Despite various studies, there is still a lack of highly effective treatments for this dual diagnosis. Therefore, ongoing research is investigating the potential of other molecules that may be effective in treating both disorders and their comorbidity. To our knowledge, no reviews or mini-reviews specifically address the use of cariprazine for the treatment of BD-SUD comorbidity. The purpose of this review is to examine and summarize the available information on the efficacy of cariprazine, recently approved by the FDA for the short-term (acute) treatment of manic or mixed episodes in bipolar I disorder, for the treatment of depressive episodes associated with bipolar I disorder (bipolar depression), for schizophrenia, and as adjunctive treatment to antidepressants for major depressive disorder (MDD) [22], in addressing the comorbidity of BD and SUD, also gathering studies that have investigated the potential of cariprazine for this comorbidity.

## 2. Cariprazine in Bipolar Disorder

Cariprazine, an atypical antipsychotic, has attracted considerable interest for its distinct pharmacodynamic properties, particularly its efficacy in the treatment of BD. It acts primarily as a partial agonist at dopamine D2 and D3 receptors, with a significant affinity for the D3 receptor [23]. This preference for D3 is thought to play a critical role in its effects on mood and cognition, as the D3 receptor is involved in the regulation of these functions [24,25,26]. Cariprazine also exhibits partial agonist activity at serotonin 1A (5-HT1A) receptors, which likely contributes to its antidepressant and mood-stabilizing properties [23,27].

In addition to its use in BD, cariprazine has demonstrated efficacy in the treatment of schizophrenia and major depressive disorder (MDD) [28,29,30]. Its ability to treat both positive and negative symptoms in schizophrenia, particularly through its D3 receptor activity, has been highlighted in several studies [28,29].

The efficacy of cariprazine in BD has been extensively studied in several clinical trials. Phase III studies have consistently shown that cariprazine significantly reduces both manic and depressive symptoms compared with placebo [30,31]. Clinical trials have consistently demonstrated the efficacy of cariprazine in the treatment of acute manic, depressive, and mixed episodes [32,33,34,35]. For example, in phase II and III randomized controlled trials, cariprazine at doses ranging from 3 to 12 mg/day demonstrated substantial reductions in Young Mania Rating Scale (YMRS) scores, with significant improvements observed as early as day 7, and these benefits were maintained throughout the 3-week study periods [33]. In addition, cariprazine has also shown efficacy in mixed episodes, where patients experience concurrent depressive symptoms during manic phases, particularly in reducing depressive symptoms, which are often difficult to treat in this context [32]. Cariprazine’s role in bipolar depression, a phase of the disorder that is often more resistant to treatment, is also promising. In several studies, cariprazine at doses of 1.5 to 3 mg/day significantly improved depressive symptoms as measured by the Montgomery–Åsberg Depression Rating Scale (MADRS) [32,35]. Importantly, the drug was effective in both pure depressive episodes and mixed episodes, in which depressive symptoms are accompanied by subsyndromal manic features [32].

In terms of tolerability, cariprazine has a generally manageable side effect profile, with the most common adverse effects being akathisia, extrapyramidal symptoms (EPSs), and nausea [32]. However, these side effects are less common at the lower doses used to treat bipolar depression than at the higher doses used to treat manic episodes [32]. In addition, cariprazine does not have the same metabolic concerns as some other atypical antipsychotics, such as olanzapine or quetiapine, which are associated with significant weight gain and metabolic syndrome [32,33]. This favorable metabolic profile makes cariprazine a more suitable option for long-term treatment, especially in patients at risk for metabolic complications [32].

Another advantage of cariprazine is its pharmacokinetic profile, which is characterized by the long half-life of its active metabolite, didesmethyl cariprazine (DDCAR) [27,36]. The half-life of this metabolite ranges from 1 to 3 weeks, which means that therapeutic levels of the drug are maintained for extended periods of time, even with occasional missed doses [27,34,36]. This property contributes to the robustness of cariprazine as a maintenance treatment by ensuring steady-state plasma concentrations, reducing the risk of relapse, and improving overall treatment adherence [32,33].

Beyond mood stabilization, recent research suggests that cariprazine may also provide pro-cognitive benefits [37]. Cognitive impairment is a major concern in BD, particularly during depressive episodes, when executive function and memory can be severely impaired [37].

## 3. Focus on Cariprazine Mechanism of Action

The pharmacodynamics of cariprazine are based on its role as a partial agonist at dopamine D2 and D3 receptors, with a notable preference for D3 receptors. As a partial dopamine agonist, cariprazine acts differently depending on the dopaminergic activity in the brain [38]. In regions where dopamine levels are elevated, such as the mesolimbic system during psychotic episodes, cariprazine reduces dopamine hyperactivity, thereby attenuating positive symptoms such as hallucinations and delusions [38]. In contrast, in areas where dopamine is deficient, such as the mesocortical pathway, cariprazine increases dopamine transmission, improving cognitive and negative symptoms [38].

In addition to its effect on dopamine receptors, cariprazine also interacts with several serotonin receptors, contributing to its broad therapeutic efficacy. It acts as a partial agonist at 5-HT1A receptors, which has been shown to have mood-stabilizing and anxiolytic effects, particularly relevant in BD and adjunctive treatment of depression [23,39]. In addition, cariprazine antagonizes 5-HT2A and 5-HT2B receptors, which helps reduce psychotic symptoms without causing severe extrapyramidal side effects [23,38]. Its weak antagonism at 5-HT2C and histamine H1 receptors is associated with a lower risk of metabolic side effects such as weight gain, sedation, and metabolic disturbances [23,38].

Studies have shown that cariprazine’s modulation of D3 receptors is particularly important in the treatment of negative symptoms [24,38]: the D3 receptor plays a role in reducing dopaminergic hyperactivity and enhancing cholinergic transmission in the prefrontal cortex, which helps improve cognitive function and emotional regulation [40,41].

Cariprazine may also potentially affect glutamatergic neurotransmission, particularly through its interactions with NMDA and AMPA receptors. It has been suggested that cariprazine may act as an NMDA receptor agonist, potentially enhancing receptor function, which is essential for synaptic plasticity, learning, and memory [42,43]. At the same time, cariprazine may have antagonistic effects on AMPA receptors, helping to regulate excitatory neurotransmission [42]. In addition, there is evidence that cariprazine may increase glutamate efflux in the prefrontal cortex, a region critical for cognitive processing, suggesting a potential role in modulating cognitive deficits [44].

## 4. Potential Impact of Cariprazine on Substance Use Disorders

The therapeutic role of dopamine partial agonists in the treatment of SUD is still relatively understudied, especially in human clinical research. In particular, the evidence from animal models is also quite limited and provides little insight into their potential efficacy. For example, a rat study demonstrated the effects of cariprazine, aripiprazole, and bifeprunox in reducing the rewarding properties of cocaine, with cariprazine and bifeprunox showing comparable efficacy that was significantly superior to that of aripiprazole [45]. Another sparse example of animal research is the D3 partial agonist CJB090, which was tested in methamphetamine-dependent rats and found to reduce methamphetamine self-administration [46].

The gap in human studies is even more pronounced. Currently, there are no randomized controlled trials or large-scale open-label studies investigating the efficacy of dopamine partial agonists for SUD. The limited evidence available comes mostly from case reports and small case series. For example, in one case report, cariprazine was shown to reduce substance use and craving and improve mood symptoms in a bipolar patient with alcohol use disorder and cocaine craving and in another bipolar patient with alcohol and cannabis abuse [47]. Another case report described the efficacy of cariprazine in improving mood and behavioral symptoms, reducing substance use, and improving overall functioning in a patient with methamphetamine-induced psychosis [48]. Further evidence from a case series involving two patients with methamphetamine dependence and psychosis showed improvements in functional outcomes and a reduction in craving following cariprazine treatment [49]. In addition, another case series highlighted the potential of cariprazine to reduce impulsivity and high-risk behaviors in patients engaging in chemsex practices, suggesting broader applications of the drug [50]. More recently, a case series [51] demonstrated the efficacy of cariprazine not only in treating psychotic and affective symptoms, but also in reducing cocaine use in a bipolar patient with comorbid cocaine use disorder.

Regarding the mechanism by which cariprazine might affect addiction and substance abuse, which, as noted above, has been demonstrated in the literature primarily through sporadic case reports, it can be speculated that its efficacy is largely due to its unique effects on D3 receptors. This feature distinguishes cariprazine from other drugs in its class, such as aripiprazole and brexpiprazole. Indeed, the dopamine D3 receptor has emerged as a key player in the neurobiology of addiction, differing from other dopamine receptors such as D1 and D2 by its specific anatomical distribution and unique functional roles [37]. D3 receptors are predominantly found in limbic regions, including the nucleus accumbens shell and ventral tegmental area, which are critical structures involved in the brain’s reward system and the reinforcing effects of addictive substances [24,37,38]. The selective expression of this receptor in areas responsible for motivation, reward processing, and emotional regulation underscores its important role in mediating the effects of drugs of abuse [37].

Chronic exposure to addictive substances such as cocaine, amphetamines, nicotine, and opioids has been shown to result in upregulation of D3 receptor expression, particularly in regions such as the ventral striatum and nucleus accumbens [52,53]. This upregulation is closely linked to the phenomenon of behavioral sensitization, a process in which repeated drug use increases an individual’s response to the substance on subsequent exposures [52,54]. This increased expression of D3 receptors in key reward-related areas is observed in both animal models of addiction and post-mortem studies of human drug users, further supporting the critical role of the receptor in the neurocircuitry of addiction [52,55].

Compounds such as SB-277011-A have been shown to reduce drug-seeking behavior, particularly when triggered by environmental cues that previously signaled drug use [56,57]. This is particularly important as cue-induced craving is a major factor in relapse [58]. D3 receptor antagonists have demonstrated efficacy in attenuating self-administration of drugs, including cocaine [59] and nicotine [60], under progress ratio schedules, which are used to measure the reinforcing strength of a drug.

Another receptor that deserves attention in the context of cariprazine’s mechanism of action is the 5-HT1A receptor (5-HT1A-R). These receptors are key components of the brain’s serotonin system, acting as autoreceptors to regulate the activity of 5-HT neurons and as postsynaptic receptors to influence activity in terminal areas [61]. Psychostimulant drugs such as cocaine, amphetamine, methamphetamine, and MDMA interact with monoamine transporters to increase extracellular levels of 5-HT, dopamine, and norepinephrine, which contribute to addictive behaviors [61]. This increase in 5-HT leads to hyperactivation of 5-HT1A-Rs, which are critically involved in both natural and drug-induced behaviors [61]. Research shows that while 5-HT1A autoreceptors tend to facilitate addictive behaviors by limiting the 5-HT response, postsynaptic 5-HT1A receptors generally work to inhibit these behaviors [61]. It is possible that this dual role may have some relevance to the therapeutic effects of cariprazine, although further research is needed to clarify this interaction.

It is also important to consider the potential role of other drugs within the same class as cariprazine in treating this comorbidity. Although there are few studies on this topic, quetiapine and risperidone have been investigated for treating alcohol use disorder, with inconclusive results [62,63,64]. Focusing on drugs more similar to cariprazine, which share partial agonism at D2 receptors, one study highlighted that patients with comorbid BD and alcohol use disorder showed symptomatic improvement when switched to aripiprazole [65]. Additionally, clozapine has shown effectiveness in managing certain symptoms of BD, such as aggression, and has demonstrated efficacy in improving SUDs involving alcohol, cannabis, and polysubstance abuse, including cocaine [66]. Clozapine’s effects on these disorders may be attributed to its diverse mechanisms, including its enhancement of GABA-B signaling, M1 muscarinic receptor agonism, and alpha-2 adrenergic receptor blockade, which increases norepinephrine levels [66]. Furthermore, clozapine reduces striatal glutamate levels, potentially mitigating the rewarding effects of substance use [66].

Regarding glutamate dysregulation in relation to SUDs, the literature has identified several mechanisms. For example, chronic drug use can disrupt glutamate reuptake, particularly through the excitatory amino acid transporter 2 (EAAT2), leading to reduced glutamate clearance from synapses and contributing to neurotoxicity and compulsive behaviors [67]. Moreover, restoring the function of the cystine/glutamate antiporter, which plays a role in regulating extracellular glutamate, using agents like N-acetylcysteine, can normalize glutamate levels and reduce drug-seeking behavior [68]. Cariprazine also influences the glutamatergic system, possibly acting as an NMDA receptor agonist and an antagonist at AMPA receptors [42,43]. However, no studies have been found in the literature specifically investigating the impact of cariprazine’s interaction with the glutamatergic system on the comorbidity of SUDs and BD.

## 5. Materials and Methods

This review was conducted by searching PubMed, Scopus, and Google Scholar databases using the keywords “cariprazine”, “bipolar disorder”, “substance use”, and “substance use disorders”. The objective was to summarize cariprazine’s mechanisms of action, focusing on those that could potentially impact the treatment of SUDs. We then aimed to review its established efficacy in BD and to investigate the presence of experimental and clinical studies that investigated the efficacy of cariprazine in the treatment of SUDs and the dual diagnosis of BD with SUD.

Preclinical and clinical studies were considered, including randomized controlled trials (RCTs), open-label studies, retrospective and prospective cohort studies, and case–control studies. Case reports and case series describing patients with comorbid BD and SUD treated with cariprazine were also reviewed. We included studies published in English until September 2024, provided they contained sufficient information to describe the type of diagnosis, the treatment administered, and the outcomes observed.

## 6. Conclusions

The landscape of psychiatric disorders is often complicated by the presence of significant comorbidities. One particularly notable comorbidity is the co-occurrence of BD and SUD, as certain psychopathological features of the former predispose individuals to developing the latter. Currently, there is a lack of robust evidence for treatments that effectively target both disorders simultaneously. Emerging data from animal models suggest that cariprazine, a promising dopamine partial agonist with potent effects on D3 receptors, may offer hope for treating this complex comorbidity. However, it is clear that the current evidence is too limited to confirm its potential utility in this dual treatment. Further studies are required, particularly in animal models, which could provide valuable insights into the mechanisms of action, such as the role of the glutamatergic system. Additionally, there is a need for more extensive research in human populations; at present, the available research is limited to case reports and small case series, underscoring the need for larger, more comprehensive clinical trials to evaluate the true efficacy of cariprazine in the treatment of both BD and SUD.
